# Analysis of factors influencing the efficacy of vagus nerve stimulation for the treatment of drug-resistant epilepsy in children and prediction model for efficacy evaluation

**DOI:** 10.3389/fneur.2024.1321245

**Published:** 2024-02-14

**Authors:** Li Su, Mengmeng Chang, Yumei Li, Hao Ding, Xiaoyu Zhao, Baomin Li, Jun Li

**Affiliations:** ^1^Department of Pediatrics, Qilu Hospital of Shandong University, Jinan, Shandong, China; ^2^Cheeloo College of Medicine, Shandong University, Jinan, Shandong, China; ^3^Department of Pediatric Surgery, Qilu Hospital of Shandong University, Jinan, Shandong, China

**Keywords:** vagus nerve stimulation, drug-resistant epilepsy, children, predictor, prediction model

## Abstract

**Objective:**

Vagus nerve stimulation (VNS) has been widely used in the treatment of drug-resistant epilepsy (DRE) in children. We aimed to explore the efficacy and safety of VNS, focusing on factors that can influence the efficacy of VNS, and construct a prediction model for the efficacy of VNS in the treatment of DRE children.

**Methods:**

Retrospectively analyzed 45 DRE children who underwent VNS at Qilu Hospital of Shandong University from June 2016 to November 2022. A ≥50% reduction in seizure frequency was defined as responder, logistic regression analyses were performed to analyze factors affecting the efficacy of VNS, and a predictive model was constructed. The predictive model was evaluated by receiver operating characteristic curve (ROC), calibration curves, and decision curve analyses (DCA).

**Results:**

A total of 45 DRE children were included in this study, and the frequency of seizures was significantly reduced after VNS treatment, with 25 responders (55.6%), of whom 6 (13.3%) achieved seizure freedom. There was a significant improvement in the Quality of Life in Childhood Epilepsy Questionnaire (15.5%) and Seizure Severity Score (46.2%). 16 potential factors affecting the efficacy of VNS were included, and three statistically significant positive predictors were ultimately screened: shorter seizure duration, focal seizure, and absence of intellectual disability. We developed a nomogram for predicting the efficacy of VNS in the treatment of DRE children. The ROC curve confirmed that the predictive model has good diagnostic performance (AUC = 0.864, P < 0.05), and the nomogram can be further validated by bootstrapping for 1,000 repetitions, with a C-index of 0.837. Besides, this model showed good fitting and calibration and positive net benefits in decision curve analysis.

**Conclusion:**

VNS is a safe and effective treatment for DRE children. We developed a predictive nomogram for the efficacy of VNS, which provides a basis for more accurate selection of VNS patients.

## 1 Introduction

Epilepsy is a paroxysmal disease caused by excessive neuronal discharges in the brain. About 70 million people worldwide suffer from epilepsy, and the prevalence of epilepsy in children is about 0.5% to 1% (1). Recurrent epileptic seizures can have a serious impact on the physical health and quality of life in children with epilepsy (2). However, 20–40% of patients still cannot control their seizures after regular antiseizure medications (ASM) treatment, known as drug-resistant epilepsy (DRE) (3). A small number of patients with clear epileptogenic focus can alleviate seizures through lesion resection (4), while there are still some patients with unclear lesions or difficulty in controlling seizures after lesion resection. In recent years, neuromodulation such as vagus nerve stimulation (VNS), deep brain stimulation (DBS), and responsive neurostimulation (RNS) have provided new options for the treatment of this group of DRE patients (5).

VNS is a method of controlling seizures by installing a chest stimulation device to stimulate the cervical vagus nerve (6). Years of clinical trials have confirmed its safety, and it has been approved for use in the treatment of DRE in children since 1997 (7). Studies have shown that VNS can reduce seizures to a certain degree in epileptic children, and improve cognition, memory, emotion, and other functions (8). However, there is limited research on the prognostic factors of VNS in children, and the selection of surgical methods mainly relies on the clinical experience of doctors, lacking clear surgical indicators. Nallammai Muthiah found that < 4-year epilepsy duration before VNS and focal motor seizures were factors associated with VNS response (9), yet no correlation was found between epilepsy duration and VNS response in J Janszky's research (10). Due to sample heterogeneity, there is no uniform conclusion on predictors of VNS efficacy, and there is still a lack of simple predictive models to predict VNS efficacy in the clinic.

In this study, we assessed the efficacy and safety of VNS by the frequency of seizures, epilepsy severity, quality of life, and adverse effects. Then we explored the possible predictors of VNS efficacy and tried to develop a prediction model for the efficacy of VNS in the treatment of DRE in children.

## 2 Methods and materials

### 2.1 Study patients

Children who underwent vagus nerve stimulation surgery at Qilu Hospital of Shandong University from June 2016 to November 2022 were selected as the study subjects. A retrospective study was conducted via their medical records, follow-up records, and epilepsy logs. All research subjects met the following inclusion and exclusion criteria. Inclusion criteria: (1) meets the diagnostic criteria for drug-resistant epilepsy: failure of adequate trials of two tolerated and appropriately chosen and used ASM schedules (whether as monotherapies or in combination) to achieve sustained seizure freedom (11); (2) age ≤ 18 years old; (3) no treatable causes were found or targeted treatment was ineffective. Among them, treatable causes include structural causes, metabolic causes, immunological causes, and age-dependent epilepsy syndrome; (4) able to complete epilepsy logs and follow up regularly for at least 6 months after surgery. Exclusion criteria: (1) patients in poor general condition; (2) patients with severe heart block and obstructive sleep apnea; (3) patients with rejection constitution or local infection; (4) patients who are unable to take regular ASMs or complete regular follow-up and epilepsy logs after surgery; (5) VNS programming procedure shut down or interrupted due to various reasons. The study was approved by the Ethics Committee of Qilu Hospital of Shandong University (Ethics Committee Approval code: No. 2017003). Before collecting patients' data, informed consent was obtained from the patients and their guardians.

Before implantation, all children with epilepsy underwent a comprehensive evaluation, including video-electroencephalogram (>8 h), magnetic resonance imaging (MRI) of brain, positron emission tomography/computed tomography (PET/CT), and cognitive function assessment.

Multidisciplinary consultation was conducted by epilepsy experts from pediatrics, neurology, and neurosurgery departments of Qilu Hospital of Shandong University, to determine suitable patients for VNS surgery. Demographic information of the patients (gender, age), etiology of epilepsy (12), onset age of epilepsy, duration of epilepsy, type of ASMs, frequency of seizures within 1 month before surgery, duration of seizures, major type of seizure, history of surgeries, MRI changes, Seizure Severity Score (NSH3) (13), and Quality of Life in Childhood Epilepsy Questionnaire (QOL-CE16) (14, 15) were collected within 1 week before surgery. Of these, seizure duration was the duration of the major seizure type that could be observed, and seizure duration of epilepsy with strings of seizures was recorded as a string of seizure duration. Intellectual disability is assessed according to the definition of the American Association on Intellectual and Developmental Disability (16).

Prognostic factors were selected based on evidence provided in literatures (6, 17–19). They were believed to be potentially clinical indicators of patient's response to VNS treatment for DRE, including: sex, seizure frequency, age at seizure onset, age at implantation, duration of epilepsy, epileptic syndromes, major type of seizures, cranial MRI changes, duration of seizure, history of hypoxic-ischemic encephalopathy, history of encephalitis, intellectual disability, history of status epilepticus, duration of VNS stimulation, types of ASMs ever used, and types of ASMs before VNS.

### 2.2 VNS implantation and programming procedure

Vagus nerve stimulator G112 (Beijing Pinch Medical Equipment Co., Ltd.), which includes a pulse transmitter, stimulation electrode, programmable control system, and magnet, was implanted through single incision surgery. After successful general anesthesia, the patient was placed in a supine position with their head tilted to the right. A transverse incision was made along the anterior edge of the sternocleidomastoid muscle at the sternoclavicular joint on the left side, and blunt separation was made along the anterior edge of the sternocleidomastoid muscle to the carotid sheath. The vagus nerve stimulation electrode and fixed anchor are wrapped around the vagus nerve trunk, and connected to the fixed electrode line. Then, separated from the lower part of the platysma muscle to the lower part of the left clavicle, forming a subfascial bag. Connected the pulse generator and fix it to the paraflavicular fascia (20–22).

Stimulator devices were turned on 2 weeks after implantation, setting the initial parameters as output current of 0.2–0.5 mA, pulse width of 500 μs, frequency 30 Hz, on-time 30 s, and off time 5 min. In magnet mode, the current is 0.1–0.2 mA higher than output current. Adjust stimulation parameters based on curative effect and adverse reactions, increase or decrease current 0.2–0.3 mA each time with remaining parameters unchanged, or increase the duty ratio if the current intensity remains unchanged.

### 2.3 Postoperative evaluation and follow-up

The frequency of seizures within 1 month before surgery (times/month) as a baseline, collected average monthly seizure frequency (times/month) during the follow-up period of the past 3 months to calculate the reduction rate of seizure frequency. According to the modified McHugh classification, postoperative patients were classified into 5 levels (class I–V). Class I is 80%−100% seizure-frequency reduction; Class II is 50% to 79% seizure-frequency reduction; Class III refers to a seizure-frequency reduction of < 50%; Class IV is only effective when using magnets; Class V indicates no improvement (23). Responders were defined as patients with a reduction of over 50% (24, 25). Frequency of seizures, type of ASMs, Seizure Severity Score (NSH3), Quality of Life in Childhood Epilepsy Questionnaire (QOL-CE16), and adverse effects were collected through outpatient follow-up, epilepsy logs, phone calls, and questionnaires at 3, 6, 12, 24, 36, and 48 months after implantation.

### 2.4 Statistical analysis

All analyses were performed using SPSS Software version 25.0 and R software. Continuous variables were expressed as the mean ± SD or median (interquartile range), while categorical data were expressed as number and percentage. Statistical analyses were two-tailed with 95% confidence intervals (CI). *P* < 0.05 was considered significant. One-way analysis of variance and paired samples Wilcoxon test were used for statistical comparison between groups. Seizure response (seizure reduction rate ≥ 50%) as the dependent variable, logistic regression was performed to screen possible influencing factors of efficacy. Based on the results of multivariate logistic regression analysis, relevant factors with *P* < 0.05 were selected to construct a nomogram for predicting the response of VNS in children with DRE. Evaluated the effectiveness of the model using the receiver operating characteristic (ROC) curve and bootstrap model. The calibration curve was employed to detect the concentricity between the model probability curve and ideal curve. The clinical benefits of our model were evaluated using the clinical decision curve (DCA).

## 3 Results

### 3.1 Demographic characteristics

A total of 45 patients were included in this study, containing 31 males (68.9%) and 14 females (31.1%). The postoperative follow-up period was 3 months to 72 months. The average age at implantation was 8.93 ± 4.30 years, with median seizure onset age of 2.58 (IQR 1.00, 5.54) years, and the average duration of epilepsy was 5.50 ± 3.51 years. Besides, a median of 4.00 (IQR 3.00, 4.00) types of ASMs had been ever used before implantation and the median number of types of ASMs used before implantation was 3.00 (IQR 2.00, 3.00). According to clinical manifestations and video-electroencephalogram (VEEG) monitoring results, the major seizure type was generalized in 29 (64.44%) patients and focal in 16 (35.56%) patients. The median duration of major seizure is 0.83 (IQR 0.33, 2.00) min. In addition, a total of 14 (31.1%) people were diagnosed with epilepsy syndrome, including 9 (20.0%) with Lennox-Gastaut syndrome (LGS), and 3(6.7%) among these LGS children were converted from West syndrome. 4 (8.9%) were West syndrome, 1 (2.2%) was Electrical status epilepticus during slow-wave sleep (ESES). Regarding the etiology of epilepsy, five (11.1%) were genetic, including one with *SCN2A* mutation, one with *KCNA2* mutation, one with *MBD5* mutation, one with *WFS1* mutation, and one with *OCRL* mutation. 11 (24.4%) were structural etiology with two tuberous sclerosis among them. One (2.2%) was immunological, and the rest 28 (62.2%) were unknown causes, which accounted for the largest proportion. 29 patients (64.4%) had intellectual disability. 16 patients (35.6%) showed structural changes on preoperative MRI, five of them didn't find clear association between structural changes and epileptic foci. Six patients (13.3%) had history of encephalitis. Six patients (13.3%) had history of neonatal hypoxia. 10 patients (22.2%) had history of status epilepticus. Four patients (8.9%) had undergone surgical lesion resection. The detailed demographic characteristics of these patients are summarized in Table 1.

**Table 1:** Demographic and clinical features of VNS patients.

**Variable**		**Overall (*N* = 45)**	**Responder (*N* = 25)**	**Non-responder (N=20)**
Sex; (n %)	Male	31	19	12
	Female	14	6	8
Epilepsy duration (years)		5.50 (3.51)	5.55(3.66)	5.44 (3.41)
VNS duration (years)		2.45 [1.26, 5.39]	4.68 [1.07, 5.39]	2.23 [1.65, 4.10]
Number of ASMs ever used		4.00 [3.00, 4.00]	4.00 [3.00, 5.00]	4.00 [3.00, 4.00]
Number of ASMs before VNS		3.00 [2.00, 3.00]	3.00 [2.00, 3.00]	3.00 [3.00, 3.25]
Seizure duration (minutes)		0.83 [0.33, 2.00]	0.50 [0.33, 1.00]	1.62 [0.29, 3.00]
Age of seizure onset year (years)		2.58 [1.00, 5.25]	3.17 [1.50, 5.83]	1.88 [0.81, 5.12]
Epilepsy syndrome; (*n* %)	Yes	14	5	9
	No	31	20	11
Major seizure type; (*n* %)	Generalized	29	12	17
Focal	16	13	3
Etiology; (*n* %)	Genetic	5	2	3
	Structural	11	10	1
	Infectious	0	0	0
	Metabolic	0	0	0
	Immune	1	1	0
	Unknown	28	12	16
MRI changes; (*n* %)	Yes	16	10	6
No	29	15	14
Neonatal HIE; (*n* %)	Yes	6	4	2
No	39	21	18
History of encephalitis; (*n* %)	Yes	6	3	3
No	39	22	17
Intellectual disability; (*n* %)	Yes	29	11	18
No	16	14	2
Operation history; (*n* %)	Yes	4	1	3
No	41	24	17
History of status epilepticus; (*n* %)	Yes	10	6	4
No	35	19	16

VNS, vagus nerve stimulation; ASM, antiseizure medication; MRI, Magnetic Resonance Imaging; HIE, hypoxic-ischemic encephalopathy.

### 3.2 Outcomes after VNS treatment

#### 3.2.1 Seizure outcomes

**Figure 1:**
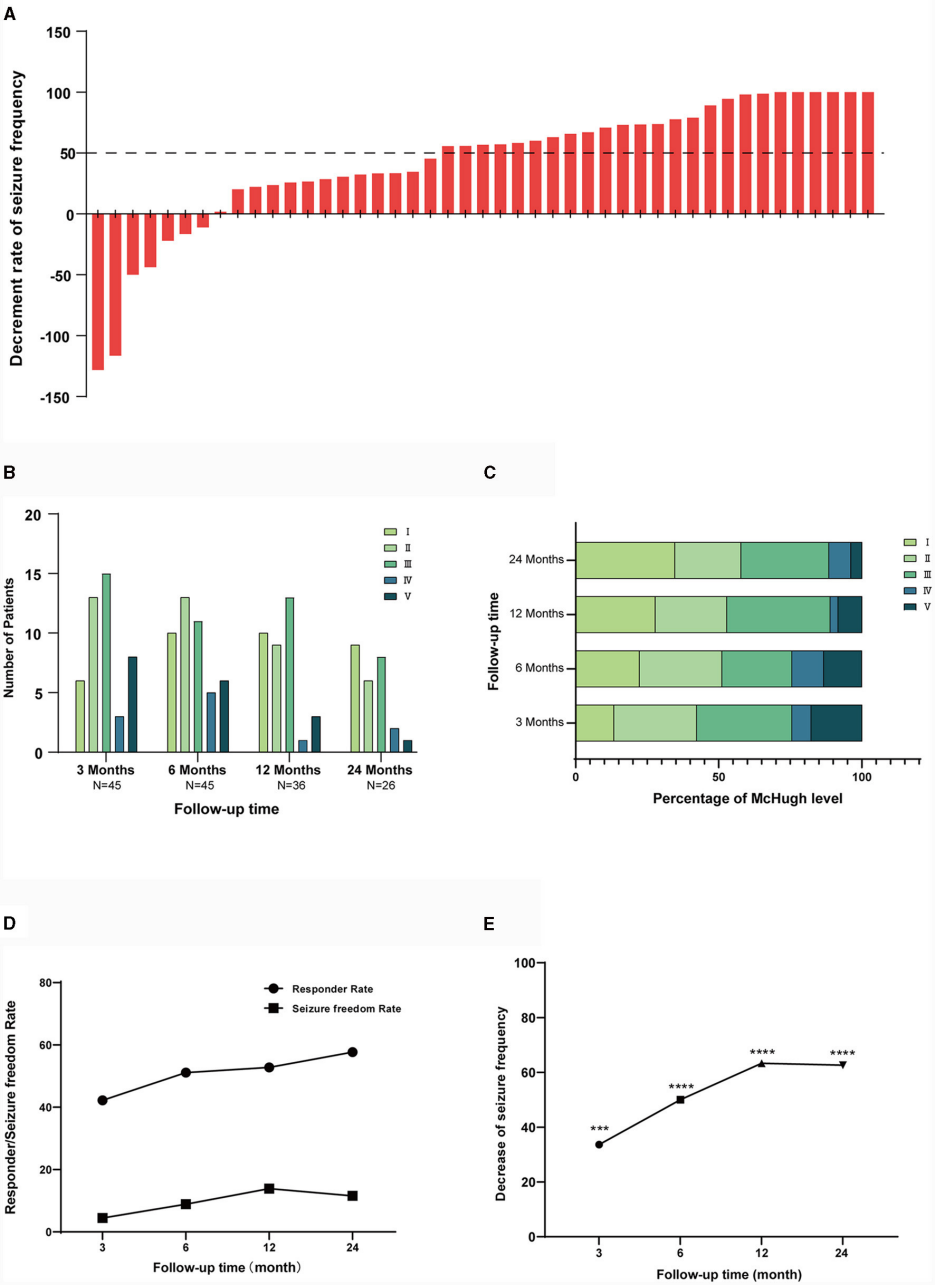
Efficacy of VNS for epilepsy. **(A)** Shows decrease in seizure frequency in all patients up to the final follow-up. **(B)** Shows the numbers of patients in each McHugh classification at different follow-up times. **(C)** Shows the percentage of people in each Mchugh classification at different follow-up times. **(D)** Shows the response rate and seizure freedom rate at different follow-up times after surgery. **(E)** Shows the mean seizure reduction rate at different follow-up times postoperatively. One-way ANOVA showing *p* < 0.05, is statistically significant in seizure frequency reduction. ***p < 0.001, ****p < 0.0001.

By the final follow-up, the median seizure decrease rate was 56.84 (IQR 24.76, 78.33), with 25 (55.6%) patients having reduction ≥50% in seizure frequency, of whom 6 (13.3%) achieved seizure freedom. According to the modified McHugh classification, 10 (22.2%) patients belonged to class I, 15 (33.3%) patients belonged to class II, 12 (26.7%) patients were in class III, 4 (8.9%) patients were in class IV, and 4 (8.9%) patients were in class V. The final reduction of seizure frequency is shown in Figure 1A.

The number of patients after 3, 6, 12, 24 months were 45, 45, 36, 26. The decline in patient numbers was due to cut-off points for data collection rather than patient attrition. During the follow-up process, reduction of seizure frequency, responder rate, and seizure freedom rate of patients gradually increased. Analysis of variance showed that there was a statistical difference (*P* < 0.05) in the reduction of seizure frequency from 3 months after implantation.

The median seizure frequency decrease at 3 months was 33.65 (IQR 0.00, 66.88), the responder rate was 42.22%, and the seizure freedom rate was 4.44%. At 6 months, the median seizure frequency decrease was 50 (IQR 1.50, 79.64), the responder rate was 51.11%, and the seizure freedom rate was 8.89%. The median seizure frequency decrease at 12 months was 63.34 (IQR 23.54, 84.44), with a responder rate of 52.78% and a seizure freedom rate of 13.88%. At 24 months, median seizure frequency decrease was 62.63 (IQR 29.77, 89.00), with a responder rate of 57.69% and a seizure freedom rate of 11.54%. Details of the McHugh classification, seizure frequency decrease, responder rate, and seizure freedom rate at different follow-up time points are shown in Figures 1B–E.

#### 3.2.2 Quality of life and seizure severity outcomes

**Figure 2:**
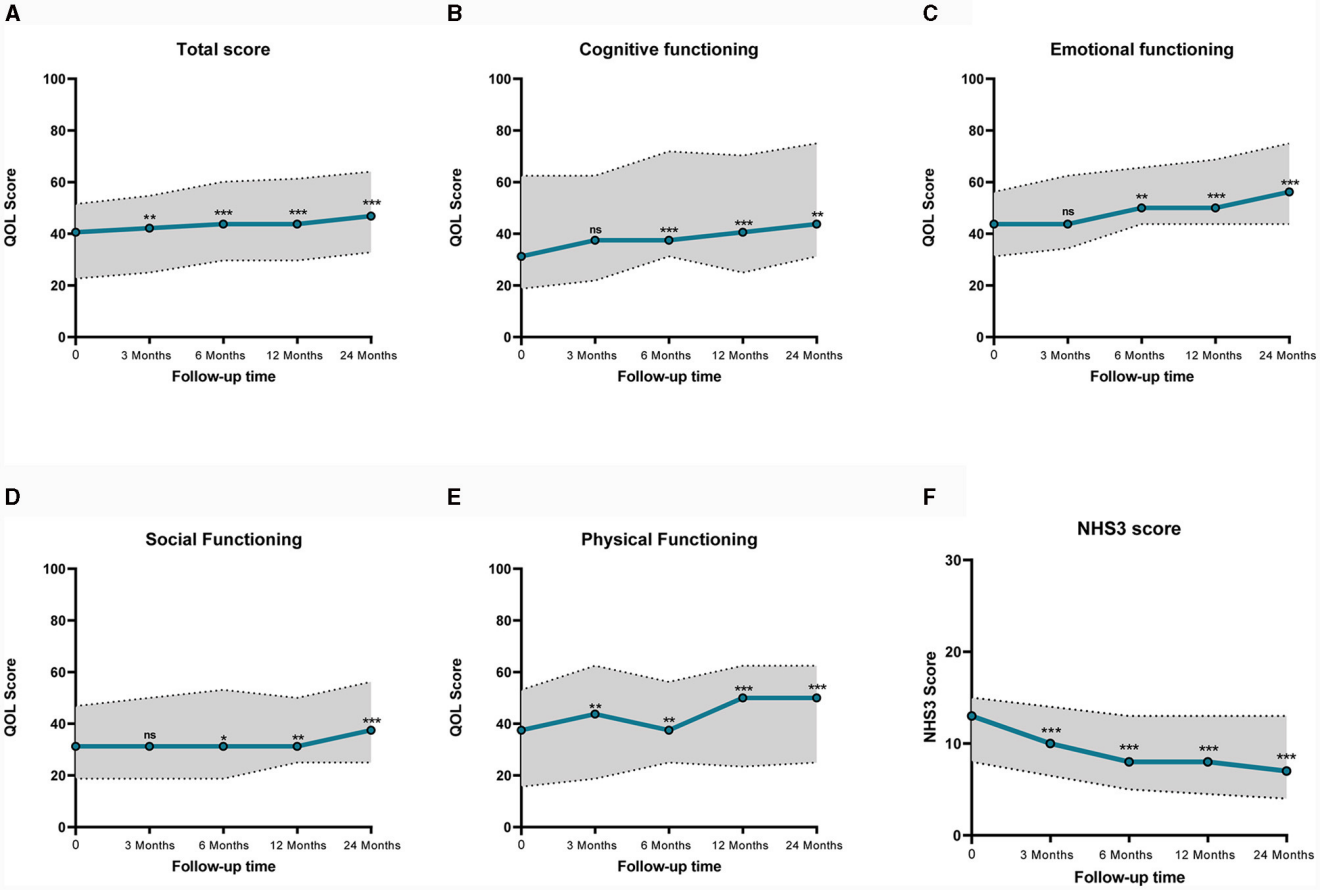
Line graph of median QOL and NHS3 scores before and after VNS. Dashed lines represent interquartile range. **(A)** Shows QOL total Score, **(B)** shows the cognitive functioning QOL score, **(C)** shows the emotional QOL score, **(D)** shows the social functioning QOL score, **(E)** shows the physical functioning QOL score, **(F)** shows the NHS3 score. QOLCE-16, Quality of Life in Childhood Epilepsy Questionnaire. NHS3, Seizure Severity Score. ns, not significant; *p < 0.05; **p < 0.01; ***p < 0.001.

The quality of life was quantitatively evaluated using the QOLCE-16, a questionnaire completed by guardians, which assessed the quality of life of children in the last 4 weeks from four aspects: cognitive functioning, emotional functioning, social functioning, and physical functioning, using a 5-point Likert scale, with final scores converted into percentages (14). Data from a total of 41 patients were available for analysis, of the remaining four patients, three had long-term carers who were unable to complete the questionnaire, and one had interrupted questionnaire follow-up. Figure 2 illustrates a line graph of QOL score, paired samples Wilcoxon test were used to compare groups. The median baseline QOL total score was 40.6 (IQR 22.7, 51.6), which was statistically different (*P* = 0.009) from 3 months after implantation. The median of baseline cognitive, emotional, social, and physical functioning scores were 31.3 (IQR 18.8, 62.5), 43.8 (IQR 31.3, 56.3), 31.3 (IQR 18.8, 46.9), 37.5 (IQR 15.6, 53.1). Except for the physical functioning, there were no statistically significant differences in cognitive, emotional, and social functioning during the first 3 months (*P* ≥ 0.05). Since 6 months after implantation, cognitive, emotional, social, and physical functioning scores all had statistically significant improvement (*P* < 0.05). After 3, 6, 12, and 24 months of VNS treatment, the improvement of QOL total score from baseline were 3.9%, 7.9%, 7.9%, and 15.5%. The detailed scores are shown in Table 2.

**Table 2:** Quality of life and epilepsy severity scores before and after VNS.

**Variable**	**Baseline (*n* = 41)**	**3 Months (*n* = 41)**	***P-*value**	**6 Months (*n* = 41)**	***P-*value**	**12 Months (*n* = 34)**	***P*-value**	**24 Months (*n* = 23)**	***P-*value**
Cognitive functioning	31.3 (18.8, 62.5)	37.5 (21.9, 62.5)	0.343	37.5 (31.3, 71.9)	< 0.001	40.6 (25.0, 70.3)	< 0.001	43.8 (31.3, 75.0)	0.001
Emotional functioning	43.8 (31.3, 56.3)	43.8 (34.4, 62.5)	0.055	50.0 (43.8, 65.6)	0.002	50.0 (43.8, 68.8)	< 0.001	56.3 (43.8, 75.0)	< 0.001
Social functioning	31.3 (18.8, 46.9)	31.3 (18.8, 50.0)	0.191	31.3 (18.8, 53.1)	0.022	31.3 (25.0, 50.0)	0.002	37.5 (25.0, 56.3)	< 0.001
Physical functioning	37.5 (15.6, 53.1)	43.8 (18.8, 62.5)	0.005	37.5 (25.0, 56.3)	0.003	50.0 (23.4, 62.5)	< 0.001	50.0 (25.0, 62.5)	< 0.001
Total score	40.6 (22.7, 51.6)	42.2 (25.0, 54.7)	0.009	43.8 (29.7,60.2)	< 0.001	43.8 (29.7 ,61.3)	< 0.001	46.9 (32.8, 64.1)	< 0.001
NHS3	13.0 (8.0, 15.0)	10.0 (6.5, 14.0)	< 0.001	8.0 (5.0, 13.0)	< 0.001	8.0 (4.5, 13.0)	< 0.001	7.0 (4.0, 13.0)	< 0.001

In addition, VNS reduced seizure severity. The NHS3 scale was applied to assess seizure severity, with a median baseline seizure severity score of 13.0 (IQR 8.0, 15.0). A significant reduction in NHS3 scores was observed, decreases in NHS3 scores at 3, 6, 12, and 24 months were 23.1%, 38.5%, 38.5%, and 46.2% (Figure 2).

### 3.3 Predictive factors and predictive models for the efficacy of VNS

#### 3.3.1 Influencing factors of VNS

Table 3 shows the results of univariate and multivariate logistic regression analyses of factors associated with VNS efficacy. Univariate logistic regression analysis found that patients with longer seizure duration had a worse prognosis than patients with shorter seizure duration (OR = 0.503, 95% CI: 0.277–0.911, *P* = 0.023), there were more responders in the focal seizure group compared with generalized seizure group (OR = 6.139, 95% CI:1.430–26.348, *P* = 0.015); patients suffering from intellectual disability were more difficult to achieve ideal results with VNS treatment (OR = 0.087, 95%CI: 0.017–0.459, *P* = 0.004). Gender, baseline seizure frequency, age at seizure onset, duration of epilepsy, epilepsy syndrome, MRI changes, history of neonatal hypoxia, history of encephalitis, history of status epilepticus, duration of VNS stimulation, and type of medication used were not statistically different between responders and non-responders (*P* ≥ 0.05). Continuous variables: seizure duration, and the categorical variables: major type of epilepsy, and intellectual disability were included in the multivariate logistic regression analyses, and all three variables were statistically significant (*P* < 0.05).

**Table 3:** Results of univariate and multivariate logistic regression analysis showing prognostic factors of VNS.

**Variable**	**Univariate analysis OR (95%CI)**	***P*-value**	**Multivariate analysis OR (95%CI)**	***P*-value**
Sex (female *vs*. male)	0.474 (0.131–1.707)	0.253	-	-
Seizure frequency ( ≤ 30 *vs*.>30 monthly)	1.556 (0.477–5.078)	0.464	-	-
Age at seizure onset (year)	1.091 (0.88–1.354)	0.427	-	-
Age at implantation (year)	1.044 (0.908–1.202)	0.544	-	-
Epilepsy duration (year)	1.009 (0.852–1.196)	0.917	-	-
Epilepsy syndrome (yes *vs*. no)	0.306 (0.082–1.141)	0.078	-	-
Major seizure type (focal *vs*. generalized)	6.139 (1.430–26.348)	0.015	17.418 (1.255–241.684)	0.033
MRI changes (yes *vs*. no)	1.556 (0.447–5.413)	0.487	-	-
Seizure duration (min)	0.503 (0.277–0.911)	0.023	0.208 (0.059–0.738)	0.015
History of HIE (yes *vs*. no)	1.714 (0.28–10.479)	0.56	-	-
History of encephalitis (yes *vs*. no)	0.773 (0.138–4.319)	0.769	-	-
Intellectual disability (yes *vs*. no)	0.087 (0.017–0.459)	0.004	0.075 (0.008–0.699)	0.023
History of status epilepticus (yes *vs*. no)	1.263 (0.302–5.275)	0.749	-	-
Duration of VNS stimulation (year)	1.143 (0.867–1.508)	0.342	-	-
Types of ASMs ever used	0.748 (0.456–1.226)	0.25	-	-
Types of ASMs before VNS	0.469 (0.191–1.153)	0.099	-	-

VNS, vagus nerve stimulation; ASM, antiseizure medication; MRI, Magnetic Resonance Imaging; HIE, hypoxic-ischemic encephalopathy; OR, odd ratio.

#### 3.3.2 Nomogram for VNS efficacy

**Figure 3:**
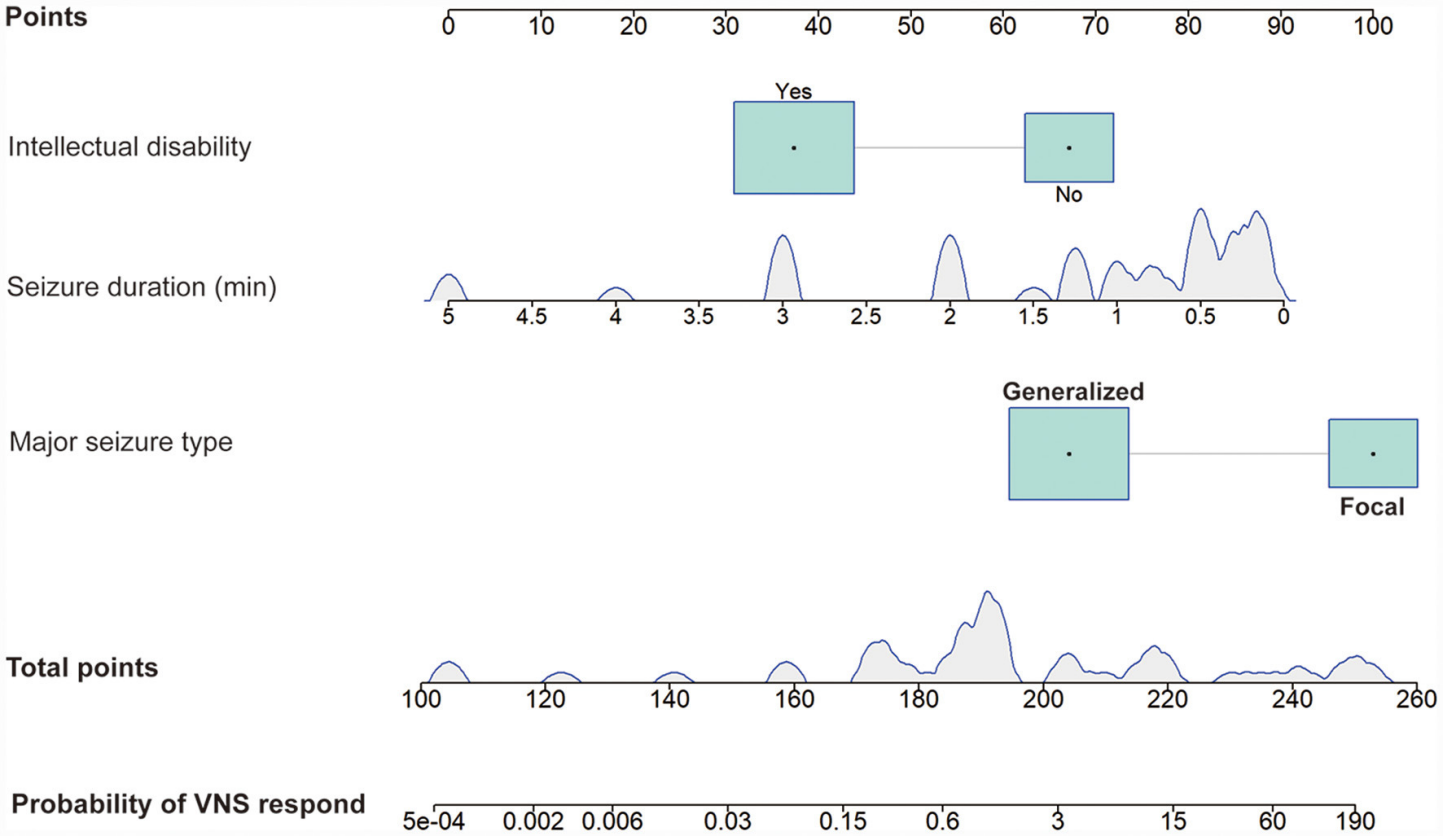
Nomogram model predicting the probability of VNS response in DRE children. The nomogram is used by summing all points identified on the scale for each variable. The total points projected on the bottom scales indicate the probability of VNS response. The density plot of continuous variables shows their distribution. For category variables, their distributions are reflected by the size of the box. VNS, vagus nerve stimulation. DRE, drug-resistant epilepsy.

A total of 16 clinical factors that may affect the efficacy of VNS were included in this study, and three variables were finally screened out: seizure duration, major seizure type, and intellectual disability. Based on the above logistic regression analysis, a predictive model for the efficacy of VNS treatment in DRE children was constructed as Logit (P) = 3.035–1.570 × [seizure duration (min)]−2.585 × [intellectual disability]+2.858 × [major seizure type]. Hosmer-Lemeshow test the model fits the observations well (χ^2^ = 11.513, *p* = 0.074). The probability that DRE children respond after VNS implantation can be estimated using the nomogram, as in Figure 3.

#### 3.3.3 Model validation

**Figure 4:**
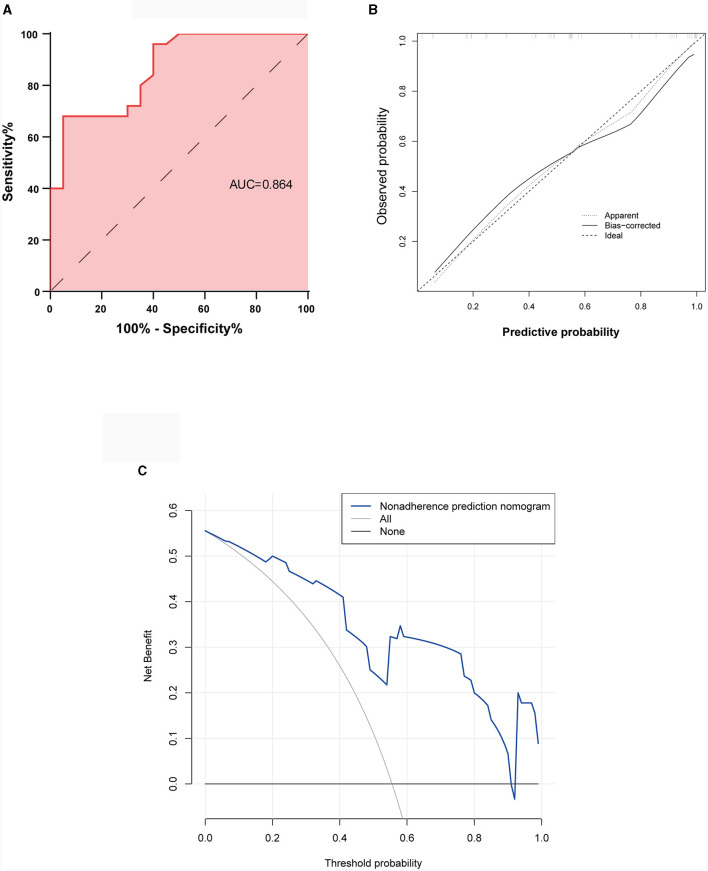
Validation of the predictive nomogram model. **(A)** Receiver operating characteristic curve. **(B)** Calibration curve for the predicted probability of the nomogram, with the x-axis representing the predicted probability and the y-axis the actual observed probability. **(C)** Clinical decision curve for the predictive model. AUC, Area under the receiver operating characteristic curve.

The performance of the nomogram was validated using ROC curve analysis, which had an area under the ROC curve (AUC) of 0.864 with a 95% CI (0.760, 0.968), indicating good diagnostic performance (Figure 4A). Bootstrap was performed for 1,000 repetitions, and the C-index of the bootstrap stepwise model was 0.837, similar to the performance of the initial predictive model. The calibration curves are shown in Figure 4B, which indicate that the nomogram may overestimate the probability of VNS response at probabilities of 0–0.5. At probabilities higher than 0.5, the nomogram may underestimate the probability. Overall, the model shows a good fit and calibration to the ideal curve. Furthermore, the decision curve analysis showed that the predictive model had a good positive net benefit when the threshold probability of VNS being effective was 0.9, suggesting that the predictive model has a favorable potential clinical effect (Figure 4C).

### 3.4 Adverse effects

Overall, VNS was relatively safe with an incidence of adverse reactions of 33.3%. No patient experienced long-term adverse effects, adverse effects were transient and reversible in most patients. Details of adverse effects are shown in Table 4.

**Table 4:** Adverse events after implantation.

**Adverse event**	**Number**	**Incidence**
Transient pain	7	15.6%
Hoarseness	4	8.9%
Coughing	1	2.2%
Nausea	1	2.2%
Subcutaneous congestion	1	2.2%
Local infection	1	2.2%
**Total**	**15**	**33.3%**

## 4 Discussion

Epilepsy is a chronic brain disease, and recurrent seizures can seriously affect the development of neurological function in children (26). The etiology of DRE in children is often more complex than that in adults (27), making it difficult to perform etiological treatment. VNS can be used in patients who are not suitable for lesion resection or who have poor prognosis after lesion resection. However, VNS is still a treatment that relies on the clinician's experience, and the clinical outcome of patients cannot be predicted preoperatively. Although many studies have explored the predictive factors of VNS efficacy in recent years, the results are not entirely consistent (18, 19, 28). Currently, there is still a lack of preoperative predictive indexes for the efficacy of VNS in the clinic.

In this study, 45 children after VNS were included and followed up for 6–73 months. With the dependent variable of whether VNS is effective in treating seizures (seizure reduction ≥50%), we analyzed 16 potential factors that may affect the efficacy of VNS in children with DRE, and identified the following independent factors: dominant seizure type, seizure duration, and intellectual disability. An easy-to-use VNS efficacy prediction nomogram was developed using multivariate analysis, which was verified to have good diagnostic performance using ROC curves (AUC = 0.864, p < 0.05) and was internally validated using bootstrap sampling methodology. In addition, the clinical decision curve (DCA) demonstrated that the predictive model had good results and potential clinical value.

We found focal seizures to be a positive factor for VNS efficacy (OR = 6.139, *p* = 0.015), which is consistent with the findings of Zhu et al. In this single central retrospective study that included 77 patients, focal epilepsy was an effective variable related to VNS (χ^2^ = 10.820, *p* = 0.004) (29). Kim et al. found that focal seizures had higher response rate than generalized seizures (16/29, 55.1% vs. 8/29, 27.6%; Pearson χ^2^ test, *p* = 0.001) (30). Nevertheless, Englot et al. found a more pronounced benefit in patients with generalized seizures compared to those with focal seizures, unknown seizure onset, and uncertainty of type (31), contrary to the findings of the present study. There is no consensus on the efficacy of different seizure forms in epilepsy. Structural lesion is one of the most common causes of drug-resistant focal epilepsy (32, 33). Recent studies have shown that VNS has neuroprotective effects in animal models of transient and permanent focal cerebral ischemia, which may be specifically related to mechanisms such as inhibition of apoptosis, mediation of angiogenesis, and protection of the blood-brain barrier (34). The protective mechanism of VNS against lesion in the brain may make it more effective against focal epilepsy than the generalized epilepsy. Inconsistent findings from clinical studies may be related to the heterogeneity of different patient groups, with different clinical decisions for patients with DRE varying from one healthcare team to another leading to this discrepancy (6).

The effect of seizure duration on patients' VNS outcomes has not been extensively studied. In this study, the mean duration of the major seizure type during 1 month preoperatively was used as a potential predictive factor on VNS outcome, and seizure duration was found to be an independent predictor of VNS outcome (OR = 0.503, *P* = 0.023). Seizures may be caused by brain lesions, and can also cause neuronal damage (35). In animal experiments, it has been found that prolonged seizure duration can lead to cell loss and subsequent synaptic network reorganization, causing damage to critical neural networks (36). The internal connections of the brain network are crucial for VNS (37), and longer seizure duration may lead to neuronal damage, causing patients to respond less to VNS than those with shorter seizure duration. Besides that, many studies have confirmed that seizure duration decreases after VNS, and a study by C Martorell-Llobregat showed that seizure duration was shorted after VNS in 88% of patients (38). We speculate that because VNS can reduce the duration of epileptic seizures, the benefits may be greater for patients with shorter duration.

Epilepsy is a chronic neurological disorder that can have an impact on brain function, because of recurrent seizures and antiepileptic drugs, children with DRE are more likely to develop cognitive impairment (39), and DRE patients with cognitive impairment have worse prognosis (40). The present study confirmed that intellectual disability would negatively affect the efficacy of VNS (OR = 0.087, *p* = 0.004). Meta-analysis of Sourbron et al. including seven studies found that VNS was less effective in epileptic children with intellectual disability (*p* = 0.18, CI 95%: 0.039–0.84) (41). The current studies have shown significant correlation between cognitive function and the efficiency of functional brain networks (42, 43). The ascending afferent neural circuit of the vagus nerve uses the solitary tract nucleus of the brainstem as a relay station and projects to the locus coeruleus-noradrenergic system, which in turn affects the limbic system, thalamus, and extensive cortical network, thereby exerting anti-epileptic effects (44). Brain network organization is one of the most powerful biomarkers for identifying VNS responders (45). Thus, more severe disruption of functional brain networks leads to reduction of VNS effectiveness, and cognitive functioning may reflect the connectivity of functional brain networks to some extent. Therefore, preoperative assessment of cognitive function plays an important role in the selection of patients for VNS surgery.

In addition, this study did not find a significant correlation between the duration of epilepsy, seizure onset age, MRI changes, and VNS efficacy (*P* > 0.05). Many studies have shown that the duration of epilepsy is a negative influencing factor for the efficacy of VNS (14, 46, 47). However, Yalnizoglu et al. found no significant differences in onset age of seizure, duration of epilepsy, implantation age, and etiology between responders and nonresponders (48). This study did not find a clear correlation between the duration of epilepsy and the efficacy of VNS (*P* = 0.917). LoPresti et al. found that brain atrophy was associated with worse VNS outcomes, while dysplastic hippocampus and chronic periventricular leukomalacia findings were found to be associated with better VNS outcomes (18). It has also been shown that patients with low seizure frequency (< 20 seizures/month) have a better prognosis (49). A retrospective analysis of 95 DRE patients with structural etiology by Xie et al. found no significant differences in gender, age of seizure onset, duration of epilepsy, number of ASMs, or specific structural etiology (50). In conclusion, despite a large number of studies exploring the clinical factors associated with VNS efficacy, different conclusions have been obtained due to patient heterogeneity, and predictors of VNS efficacy need to be further investigated. In this study, a simple nomogram was created for the first time to predict the efficacy of VNS in DRE children. The nomogram may help clinicians to select patients who are more suitable for VNS surgery. Larger clinical trials are needed to further validate the performance of this predictive model.

## 5 Conclusion

This study verified that VNS is an effective treatment for DRE in children, with a response rate of 55.6% at the final follow-up. The quality of life and seizure severity of children improved significantly after VNS treatment. Patients with focal seizures, short duration of seizures, and absence of intellectual disability are more suitable for VNS.

## Data availability statement

The raw data supporting the conclusions of this article will be made available by the authors, without undue reservation.

## Ethics statement

The studies involving humans were approved by the Ethics Committee of Qilu Hospital of Shandong University. The studies were conducted in accordance with the local legislation and institutional requirements. Written informed consent for participation in this study was provided by the participants' legal guardians/next of kin.

## Author contributions

LS: Data curation, Investigation, Writing—original draft. MC: Software, Visualization, Writing—original draft. YL: Data curation, Writing—review & editing. HD: Data curation, Writing—review & editing. XZ: Data curation, Writing—review & editing. BL: Conceptualization, Methodology, Writing—review & editing. JL: Conceptualization, Methodology, Writing—review & editing.
